# Genome-scale patterns in the loss of heterozygosity incidence in *Saccharomyces cerevisiae*

**DOI:** 10.1093/genetics/iyac032

**Published:** 2022-02-25

**Authors:** Hanna Tutaj, Adrian Pirog, Katarzyna Tomala, Ryszard Korona

**Affiliations:** Institute of Environmental Sciences, Jagiellonian University, 30-387 Cracow, Poland

**Keywords:** heterozygosity, rate of LOH, chromosome arm, centromere, telomere, mitosis, starvation, environmental stress

## Abstract

Former studies have established that loss of heterozygosity can be a key driver of sequence evolution in unicellular eukaryotes and tissues of metazoans. However, little is known about whether the distribution of loss of heterozygosity events is largely random or forms discernible patterns across genomes. To initiate our experiments, we introduced selectable markers to both arms of all chromosomes of the budding yeast. Subsequent extensive assays, repeated over several genetic backgrounds and environments, provided a wealth of information on the genetic and environmental determinants of loss of heterozygosity. Three findings stand out. First, the number of loss of heterozygosity events per unit time was more than 25 times higher for growing than starving cells. Second, loss of heterozygosity was most frequent when regions of homology around a recombination site were identical, about a half-% sequence divergence was sufficient to reduce its incidence. Finally, the density of loss of heterozygosity events was highly dependent on the genome’s physical architecture. It was several-fold higher on short chromosomal arms than on long ones. Comparably large differences were seen within a single arm where regions close to a centromere were visibly less affected than regions close, though usually not strictly adjacent, to a telomere. We suggest that the observed uneven distribution of loss of heterozygosity events could have been caused not only by an uneven density of initial DNA damages. Location-depended differences in the mode of DNA repair, or its effect on fitness, were likely to operate as well.

## Introduction

Mitosis evolved to enable a cell to divide into 2 cells with identical content and arrangement of genetic elements. Any differences between resulting genomes signal failures in this process and provide an opportunity to learn how the typically observed genetic stability is achieved. In diploids, one of such inadvertent genetic changes is the loss of heterozygosity (LOH), that is, a failure to transmit both alleles to a descendant cell when 1 of 2 different alleles is replaced by the other one (homozygosity) or lost altogether (hemizygosity). The study of LOH in mitotically dividing diploid cells of the budding yeast has yielded a wealth of information about the behavior of chromatids at the time of replication, repair, or segregation (Pâques and Haber 1999; Stirling *et al.* 2011; Symington *et al.* 2014). That research was centered on uncovering the molecular mechanisms underpinning the process of mitosis. A broader perspective, with LOH being a genetic phenomenon of serious consequence for the evolution of this species, was underappreciated. One reason was that the wild strains of yeast were expected to undergo frequent selfing (within an ascus or within an initially haploid but homothallic colony). This would greatly accelerate the exposition of recessive deleterious mutations to the action of purging selection ensuring evolutionary advantage, and hence high prevalence, of sexual and mostly homozygous strains (Mortimer *et al.* 1994; Knop 2006). This notion has not been abandoned after the early reports of its possible inadequacy (Kelly *et al.* 2012; Magwene 2014). Only after the completion of large and comprehensive surveys of strains derived from different geographical and ecological locations, an abundance of heterozygosity, and hence the potentially important role of mitotic LOH have been finally recognized (Peter *et al.* 2018; Fischer *et al.* 2021). Considering other and very different populations, those of human cancer cells, the meaning of LOH as a critical factor in the progression of tumors has been early understood and never questioned (Knudson 1971). Indeed, recent analyses have demonstrated how unexpectedly important it can be: for about 50 most commonly found cancer driver genes, biallelic mutations (resulting from LOH) have been identified for all of them and typically as prevailing over monoallelic ones (The ICGC/TCGA Pan-Cancer Analysis of Whole Genomes Consortium 2020). In sum, the rationale for studying LOH in *Saccharomyces* *cerevisiae* is not only still valid but strengthening. The goal would be to elucidate the population genetics of this particular species but also to maintain its role as an outstanding model in research on molecular evolution of eukaryotes.

A classic approach in the study of LOH has been to use strains with heterozygous loci in which a recessive allele renders an occurrence of a conspicuous trait when its dominant counterpart goes absent. Among the most popular ones, there have been those providing resistance to specific chemicals (*CAN1/can1*, *URA3/ura3*), changing colony color (*ADE2/ade2*, *MET15/met15*), or restoring mating ability (*MAT***a***/MATα*). Counts of colonies with an original and altered phenotype have been then used to estimate the rate of LOH. The nature of chromosomal events (such as cross-over, local conversion, break-induced repair, nondisjunction, truncation) could have been also inferred if planned arrangements of specific genetic elements were used (Andersen *et al.* 2008; Symington *et al.* 2014). As one or very few loci have been used in a particular study, estimates of a genome-wide LOH rate had to rely on extensive extrapolations.

Analyses of an entire genome have been made possible by high throughput sequencing technologies. Regarding LOH in yeast, a typical start point is to derive a diploid strain descending from 2 haploids with densely distributed single nucleotide polymorphisms so that even relatively short fragments of chromosomes can be ascribed to one of the ancestors. Replicate populations of such hybrids are then propagated as separate mutation accumulation (MA) lines through periodic reduction of every population to a single cell (actually, a single colony-forming unit which makes the procedure less stringent). Subsequent comparisons with an ancestral strain can reveal spontaneous rearrangements or losses of segments of very different sizes, from a whole chromosome to about a single gene (Tattini *et al.* 2019; Loeillet *et al.* 2020; Pankajam *et al.* 2020; Sui *et al.* 2020). In such studies, the number of detected LOH events tends to be relatively small as it is limited by the number of propagated cell lines and sequenced genomes. Periodic reductions to single colony-forming units are meant to fix new mutations, but such moments are separated by about 2 dozen divisions within an expanding colony when selection can operate. Altered karyotypes would be mostly removed but positive selection of them cannot be excluded (Sheltzer and Amon 2011; Plech *et al.* 2014; Shapira *et al.* 2014; Deregowska *et al.* 2015; Laiba *et al.* 2016; Zhu *et al.* 2016; Kaya *et al.* 2020). Therefore, it appears worthwhile to compare results of the MA studies with those obtained in a radically different manner.

**Fig. 1.**
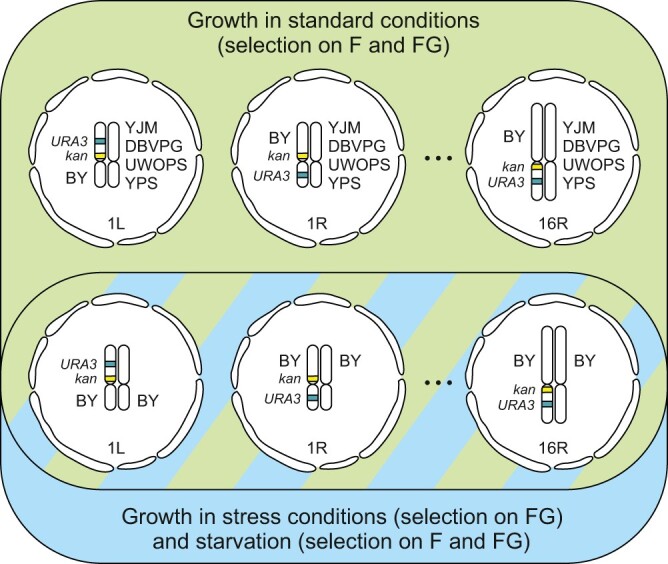
Strains, environments, and selective media. Each of 32 chromosome arms (from 1L to 16R) of the BY strain was marked with *kan* (geneticin resistance) inserted close to a centromere and *URA3* (sensitivity to 5-FOA) inserted close to an arm midpoint. Such marked haploids were used to form 1 set of homozygous and 4 sets of hybrid diploid strains. Replicated cultures of those were propagated under standard or stress conditions and then LOH mutants were scored on either F (5-FOA) or FG (5-FOA plus geneticin) selective media.

Our goal was to spot LOH events possibly immediately after they appeared, score them in large numbers, and do it in such a way that the LOH contribution of small chromosomes to whole-genome estimates would not be eclipsed by that of large ones. A start point was to put a counter selectable marker (*URA3*) at the central region of every arm and a positively selectable one (*kan*) in the proximity of a corresponding centromere. After brief propagation, necessary to obtain multiple cultures of the same clone, a screen was applied to detect colonies lacking *URA3* and thus exhibiting LOH. The concurrent presence of *kan* indicated recombination between the markers. A set of such marked strains was used in several genetic and environmental arrangements (Fig. 1). This approach enabled us to concentrate on questions that had not been straightforwardly asked or compellingly answered in former studies.

## Materials and methods

### Growth media and reagents

Synthetic complete medium (SC), without uracil when selecting for Ura^+^ transformants and temperature 30°C were applied as standard growth conditions. Two basic selective media contained 5-FOA (1 g/l), either solely or together with geneticin/G418 (400 mg/l). Test environments were based either on SC (with temperature shifted to 37°C or 40 g/l of NaCl added) or on rich YPD medium (with temperature shifted to 24°C or 32°C or kept at 30°C but with glucose replaced with 3% glycerol). Sporulation was induced by growing cells overnight at 30°C on GNA agar (5% glucose, 3% Difco Nutrient Broth, 1% Difco Yeast Extract) and then inoculating them thinly to liquid 1% potassium acetate with 0.005% zinc acetate and required nutrients at one-fourth of standard levels for about a week of incubation at 25°C with shaking.

### Collection of 32 middle-arm marked strains

*URA3* gene of *S.* *cerevisiae* from the pRS416 plasmid was embedded into an expression cassette, *MX4*, and used to make targeted inserts into haploid strains of *S. cerevisiae*, BY4741 or BY4742. Insertions were guided by ∼45 bp flanking fragments homologous to genomic DNA sequence on 30 of 32 arms of yeast chromosomes. Target sequences were selected as possibly closest to the midpoint between a centromere and a telomere and not overlapping with known coding or regulatory sequences. In particular, we preferred spots located immediately after a terminator of an essential gene, because the viability of transformants signaled that no conspicuous damage was done. Successful PCR amplification, extending over the sites of expected recombination, was regarded as an indication of correct insertion. The 2 remaining constructs, the left arm of chromosome IX and the left arm of chromosome XIV, were created by replacing the YKO *kanMX4* cassette, that was present as a gene replacement close to the middle of the arm, with a *URA3MX4* cassette. A list of insertion positions and primers used are provided in Supplementary Table 1.

The described above constructions yielded 32 distinct strains marked with a single *URA3* marker. There were in fact 16 pairs of strains, each pair represented by a left (L) and right (R) arm of the same chromosome. For each pair, we inserted a selectable marker possibly close to a centromere. As we were cautious not to introduce new phenotypes and especially not to destabilize centromeres, we browsed the YKO collection for deletions (which were in fact replacements with the *kanMX4* cassette) that resulted in no visible phenotype, were close to a centromere, and were transcribed in a direction opposite to that centromere (the selected positions are listed in Supplementary Table 1). Each selected YKO deletion strain was then mated with each of the 2 strains having *URA3* on the same chromosome, 1 on the left, and 1 on the right arm. Resultant diploids were sporulated and used in tetrad analyses in which 32 double marked haploids, with *URA3* and *kan*, were obtained. The haploids were tested for normal growth, i.e. typical for the BY background strain, and thus did not show signs of damages introduced by the described manipulations. They were then mated with 5 genetically distant haploid strains of stable mating types: BY4741, UWOPS05-227.2, YJM975, YPS606, and DBPVG6044 (Cubillos *et al.* 2009). The final collection of 160 (32 × 5) double-marked strains was deep-frozen and saved for assays described below.

DNA sequence and genome annotations were extracted from the *Saccharomyces* Genome Database. Positions of genetic elements previously found as potentially affecting LOH (see *Discussion*) were identified with an SGD feature Genome Snapshot (as of May 2021), the Tandem Repeats Database (Gelfand *et al.* 2007), and (Mancera *et al.* 2008).

### Collection of 23 strains with variably positioned markers

Haploid BY4741 (*MAT***a**) and BY4742 (*MATα*) deletion strains were used. Genomic segments of about 40 kb not containing any repetitive elements, especially those identified as enhancing the rate of LOH, were selected. The segments were also required to end, on both sides, at an ORF deletion that did not result in destabilization of the genome or serious growth defect. For every segment, a pair of deletion strains with opposite mating types was taken out of the YKO collection. For every pair, the *kan* marker closer to its centromere was retained while the other replaced with *URA3*. The 2 strains were mated and the resulting diploids were sporulated, spores separated, and haploids carrying both markers were secured. They were then mated with appropriate BY haploids so that standard BY4743 diploids (designated here as BY/BY) could be derived and used in LOH assays. Supplementary Table 2 lists positions of the selected ORFs.

### Selection for LOH mutants

Thawed samples of the collection of 32 double-marked strains were used to inoculate 200 µl of SC aliquots arrayed on plates with 96 flat-bottom wells. After 48 h of incubation without agitation, these initial microcultures were serially diluted so that no more than 100 cells were present per well in 200 µl of fresh medium. This was a start point for the accumulation of LOH mutants. Such inoculations were done independently for at least 30 replicate microcultures for every double marked strain. (There were 3 ordered arrays of 32 strains per each of 10 or more plates.) The LOH-accumulating cultures were allowed to reach the stationary phase (from 3 days for SC to 6 days for YPG). They were then diluted 1:10 in water. Ten microliters of aliquots were transferred onto SC agars supplemented with 5-FOA plus G418 (all assays) or 5-FOA only (selected assays). Plates were incubated and photographed after 24, 48, and 72 h. LOH mutants emerging on the selective agars were sufficiently discernible to a human eye making manual counting feasible. In addition to this assay, devised to identify only the LOH mutants, another one, devised to estimate densities of entire cultures, was carried out concomitantly. To this end, the LOH-accumulating cultures were diluted further and 10 µl samples of the final dilution, after sonication, were transferred on nonselective SC agar. They were calibrated to contain no more cells that could be individually discerned and counted (after forming colonies), usually 20–40. These counts served to estimate the number of cells exposed to the selective agents. The collection of all 160 marked strains was used in the described above screens after accumulating LOH mutants in the standard SC medium. The 32 BY/BY strains accumulated the mutants also in the mentioned earlier environments in independently inoculated replicate cultures (Fig. 1).

The collection of 23 strains was grown in the same way but the final replicate mutation-accumulation cultures were of 20 µl aliquots kept in round-bottom 96-well titration plates. There were 20 replicate cultures for each strain tested. The wells were thoroughly washed up and the entire content of a single well was overlaid on an individual agar plate with 5-FOA and geneticin. Counts of colonies were obtained by visual inspection.

### Estimation of the LOH rate

In the experiments involving the 32 strain set, only a sample of 1/200 of every replicate LOH-accumulating culture was exposed to selection to reveal the number of mutants present in it. We had to take such small samples because the frequency of LOH mutants was high and larger samples could not be processed in a high throughput manner, as too many mutant colonies would show up at every deposition spot. Proceeding from colony counts to rate estimates can be done in several ways. This could involve approximation of the number of mutant-free replicate cultures or calculating average mutant frequencies (Foster 2006). We reasoned that our data are better suited for the latter. Namely, a mean frequency of mutants within tested populations μ can be approximated as *μ* = *f/*ln(*N*μ), where *N* is the population size and *f* is the frequency of mutants (Drake 1991). To estimate *f*, we used our own measure of the central tendency in replicate counts. We ln-transformed nonzero counts, averaged them, and used this average as an exponent over *e* in a reverse transformation. The resulting value was then multiplied by the proportion of nonzero counts among all replicate cultures (to account for the zero counts). We attempted to avoid biases introduced by exceptionally large counts that would occur if an arithmetic average was used. We did not use a median because sometimes, although rarely, more than half of replicate counts were nulls. To provide examples of our intermediate estimates, for the SC growth medium, BY/BY strain and FG selective agar, the value of *N* was 2.2 × 10^7^ while *f* varied from 7.8 × 10^−6^ for the short arm of chromosome VI to 7.4 × 10^−4^ for the long arm of chromosome XII. To find 95% confidence limits of the final estimate of *μ*, we applied a standard bootstrap procedure in which counts were drawn at random, and then *μ* was calculated, 10,000 times for every estimated *μ*.

For the assays involving the collection of 23 strains, the FluCalc program (based on maximum likelihood calculations) was used; it provided both central tendencies and confidence limits of the LOH rate (Radchenko *et al.* 2018).

Statistical tests were conducted using the base R package with the addition of aov (2-way ANOVA with post hoc Tukey multiple comparisons), plyr (Pearson’s product moment correlation) as well as lm, scaled data, and step (multiple regression analyses). The drawing of plots was initiated with ggplot2 (Wickham 2016) and then finished with CorelDRAW 2019.

### LOH under starvation

Replicated stationary phase cultures of the 32 BY/BY single-arm marked strains were diluted 1:10 in water to create 200 µl starving cultures kept in 96-well flat-bottom plates. Plates were put into plastic bags (to contain evaporation but allow for some ventilation) and incubated at room temperature in the dark. The cultures were kept for the first 3 days undisturbed to condition them in the new environment. After that, an initial sampling was done by transferring 10 µl samples onto 2 selective agar plates, with 5-FOA and 5-FOA + G418, to detect LOH mutants. To estimate the density of entire cultures, samples of them were diluted appropriately so that possibly large but countable numbers of colonies were obtained on solid (nonselective) SC medium. Sampling was repeated after 7, 14, and 21 days of starvation.

To interpret correctly the described above experiment, we needed to know: (1) what were the relative plating efficiencies on the selective and nonselective media; (2) if there were any potential differences in sensitivity to starvation between the LOH mutants and wild types; (3) did uracil auxotrophy (in LOH mutants) have any impact on viability or plating efficiency. To answer these questions, we assembled a set of strains consisting of individual single-arm marked BY/BY strains accompanied by 3 LOH mutants derived from each of them (selected at random). An unmarked BY/BY progenitor strain (as it lacked *URA3* but did not undergo LOH) was also included. These strains were conditioned and starved in the way described above. They were sampled in a way enabling exact counts of colonies emerging on agar surfaces, that is, we followed the procedure developed for counting colonies on the nonselective SC medium. However, the samples were transferred on both the nonselective medium and the double selective medium, 5-FOA + G418.

### RNA extraction and transcriptome analyses

BY4743 (designated also as BY/BY in this paper), which is diploid and isogenic to our collection strain, was used to assay gene expression in several environments. Cultures were prepared in the same way as those used for LOH accumulation (200 µl aliquots in titration plates) but harvested in the advanced growth phase instead of the stationary phase. Two replicas of the tested strain were independently initiated and propagated in every tested environment. Total RNA was extracted with the RiboPure RNA Purification Kit. Libraries preparation and PE 150 sequencing was carried out by Novogene. About 20 mln read pairs were obtained per sample. Quality control of the reads was performed with fastQC v0.11.9 (Andrew 2010). Reads were then aligned to Ensembl release 100 *S.* *cerevisiae* genome with Hisat2 v2.1.0 (Kim *et al.* 2015). The resulting alignment files were sorted and indexed with samtools (1.9). Transcript quantification was performed with cuffquant/cuffnorm v2.2.1 (Trapnell *et al.* 2012). Gene count data normalization and differential expression analysis were performed in the EdgeR exact test (Robinson *et al.* 2010).

## Results

### LOH was more frequent in homozygotes than hybrids

**Fig. 2.**
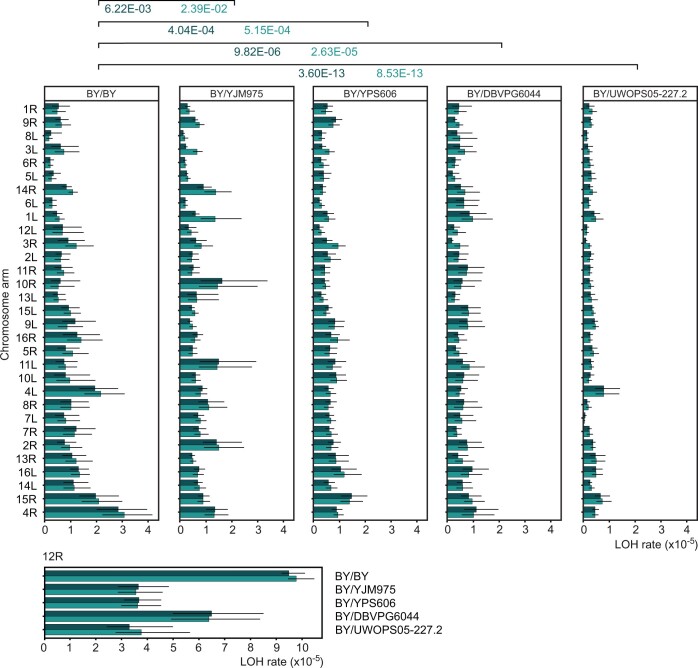
Rate of LOH events per cell division estimated for each chromosomal arm. Arms are ranked from the shortest (top) to longest (bottom) distance between the markers, the right arm of chromosome XII is depicted separately as an outlier. The hybrids are ordered according to an increasing genetic distance to BY. Dark bars show the rate and 95% confidence limits obtained from counts on the FG medium (recombination between a centromere and midpoint of every arm); light bars refer to the F medium (both recombination and chromosome loss). The top 31 arms in 5 strains were compared in a 2-way ANOVA, full results are presented in Supplementary Table 4. In this figure, only probabilities of the type I error associated with rejecting the null hypothesis of the equal rate of LOH in the homozygous and one of the hybrid strains are shown. LOH estimates with confidence intervals are listed in Supplementary Table 3.

We estimated the rate of LOH for 1 set of homozygotes and 4 sets of hybrids, each composed of 32 strains (Fig. 1). Cells were propagated under standard conditions and then LOH mutants were screened on 2 selective media, 1 with 5-FOA plus geneticin (FG), and 1 with 5-FOA only (F). The FG medium selected for the presence of *kan* but loss of *URA3.* On the F medium *URA3* was necessarily absent but *kan* was either present or absent. Estimates of the rate of LOH per single cell division are presented in Fig. 2 and Supplementary Table 3. As expected, LOH was detected more frequently when the markers resided on longer chromosomal arms. F and FG did not differ significantly, suggesting that the loss of chromosomes (detectable on F) was not a dominant source of LOH. In these 2 respects, all 5 genetic backgrounds yielded similar results. However, there was 1 and crucial difference between the 5 strains. The rate of LOH was highest in the homozygous BY/BY and it tended to decrease as the genetic distance between strains constituting a hybrid increased. The latter dependence is demonstrated by the pairwise comparisons reported in Fig. 2 and, in more detail, in Supplementary Table 4.

We then sought to combine the reported above single-arm estimates into overall per-strain estimates. We did it only for the data obtained with the FG medium. The reason was that the loss-of-chromosome mutants were in a clear minority in our study and still rarer in the MA studies with which we compare our results. To start, we multiplied our original estimates by 2 as our assay uncovered only an estimated half of LOH events. (This is because a LOH-associated division of a +/− cell produces +/+ and −/− or +/+ and +/+ or −/− and −/− cells; 5-FOA allows for growth only when there is no active allele. Similarly, an expected half of chromosome losses is not seen.) We then summed the rates of LOH obtained for segments bordered by the *kan* and *URA3* markers. In the case of BY/BY, the rate of LOH events per 1 cell division when accounting for all 32 segments was 7.46 × 10^−4^. It equaled 5.53 × 10^−4^ after excluding 12R, which hosts the rDNA cluster where homologous recombination is unusually intense. The latter estimate appears more representative for the LOH rate typically encountered in the yeast genome. Considering that the 31 segments covered together 45.3% of the length of all 16 chromosomes, an extrapolated estimate for the whole genome amounted to 1.22 × 10^−3^ of LOH events per 1 division of a BY/BY cell. For YJM975, YPS606, DBPVG6044, and UWOPS05, it was 8.91 × 10^−4^, 8.17 × 10^−4^, 7.31 × 10^−4^, and 4.02 × 10^−4^, respectively. Note that these calculations were based on an assertion that the LOH events were distributed with equal density in the inward and outward parts of chromosomes.

### Shorter chromosome arms had a higher average density of LOH

**Fig. 3.**
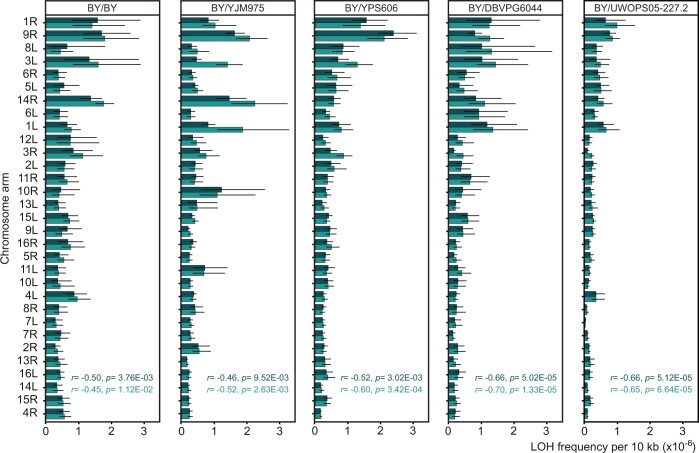
Average density of LOH events per 10 kb per cell division. (These are rates reported in Fig. 2 but divided by the physical distance between markers.) Pearson’s coefficients of correlation between the LOH density and the rank of chromosome length are shown together with the corresponding type I error estimates (not corrected for multiplicity of comparisons). LOH estimates with confidence intervals are listed in Supplementary Table 3.

Figure 3 shows average densities of LOH per 10 kb calculated individually for inner halves of arms without extrapolations on the unstudied distal halves. A clear pattern, supported by highly significant results of statistical tests, is easy to see: the shorter the arm the higher the rate of LOH events per unit length of the chromosome, irrespective of whether the FG or F medium is considered. It was found in 5 independently prepared and executed experiments, with 1 homozygous and 4 hybrid strains. The trend was not only regular but also strong, the LOH density was several-fold higher on the shortest arms than on the longest ones.

### LOH was affected by repetitive elements but not intensity of transcription

We reasoned that the observed variation in the rate of LOH could be related to the burden of repetitive elements in the studied parts of chromosomes or to the intensity of metabolism affecting them (such as transcription). We selected several environments known to activate different sets of genes, resulting in pronounced shifts in the transcriptional intensity across chromosomal arms. Both the presence of repetitive sequences and the abundance of transcripts can be determined most straightforwardly for homozygotes, so we restricted our study to BY/BY. We cultured the collection of 32 strains in several media—SC at 30°C, SC at 37°C, SC at 30°C with NaCl, YPG at 30°C, YPD at 24°C, and YPD at 32°C—and scored colonies emerging on the FG selective medium. We then calculated arm-specific rates of LOH events per 10 kb (analogous to that shown in Fig. 3) in each of these environments. Such obtained rates of LOH were then analyzed as a variable dependent on several genetic determinants. Among them, there were densities (per 10 kb) of different repetitive elements present in every analyzed between-marker stretch of DNA. We also added a determinant variable that was obtained in this study. Specifically, we extracted mRNAs from cells grown in every environment. We then estimated how intense the transcription per unit length of a given chromosomal arm in every environment was (% of cellular mRNA produced within a given segment).

**Fig. 4.**
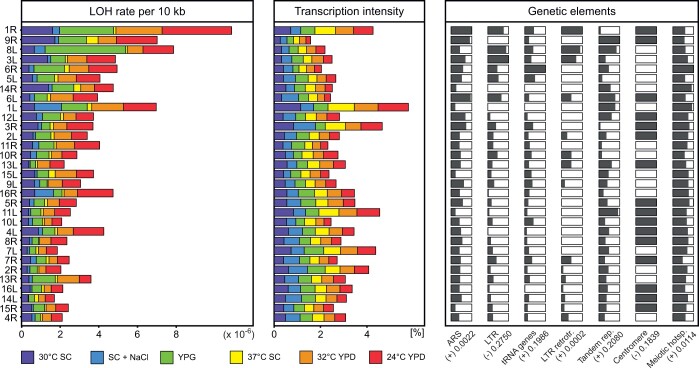
Environmental and genetic determinants of the density of LOH events (per 10 kb per cell division). Estimates of the dependent variable (LOH density) are collected in the left graph (see Supplementary Table 6). One potential determinant of LOH density is the intensity of transcription (amount of mRNA per 10 kb of a tested region). The length of bars illustrates the relative intensity of transcription calculated separately for each environment. The right panel refers to genetic elements as potential determinants. Relative abundance of a particular genetic element is shown as a gray field within a bar, an arm with the highest density is full gray. The numbers under it refer to the type I error associated with coefficients of multiple regression, signs in parentheses show the direction of associations.

Figure 4 shows how the dependent variable (LOH per 10 kb) related to the determinant variables in a joint analysis of multiple regression (Supplementary Table 5). The rate of LOH changed in nonstandard conditions; in particular, it was considerably lower in SC at 37°C and SC at 30°C with NaCl. However, the pattern of negative relation between the LOH density and arm length was present in all environments tested, underscoring the generality of this finding. No comparably clear conclusion emerged from an analysis of the intensity of transcription. Note that patterns of expression were considerably different between environments. Within a single environment, the intensity of transcription was often several-fold higher on some chromosome arms than on others but this variation appeared to be statistically neutral for LOH. Indeed, even a quick visual inspection of Fig. 4 indicates that the pattern of decreasing LOH rate with an increasing arm length (left panel) is not reflected in the relative transcription intensity (middle panel). Regarding the relation between the density of LOH and the density of selected genetic elements, the results were mixed. The selection of elements was based on earlier studies that, sometimes though not always, had linked them to LOH (see *Discussion*). There was a considerable variation in the distribution of those elements on the studied chromosome segments. However, associations were not always statistically significant (Fig. 4). Statistically significant and positive associations with LOH were found for ARS, LTR retrotransposons (including inverted Ty elements), and meiotic hotspots.

### LOH incidence was affected by the proximity of telomeres and centromeres

As reported above, we found that LOH was distributed more densely on short chromosomal arms (Fig. 3). Those arms also tended to have a higher density of the genetic elements positively associated with LOH (Fig. 4). It was therefore unclear whether the length of an arm would remain such a strong (if any) determinant of LOH if those elements were absent. A way to circumvent this problem would be to show that not the shortness of an arm in itself but the proximity of its centromere or telomere was actually critical. To test this conjecture, we looked for stretches of DNA that would be appropriately long and completely free of the reported above destabilizing elements. Such regions were scarce, absent on several arms, while occurring only once or a few times on others. This constrained our choice but did not invalidate our objective as not the identity of a chromosome but the impact of the distance to its ends was of interest. We also wanted the target regions to be flanked by genes that have no strong phenotypic effects, especially those related to genome stability. The reason was that we planned to have one of them replaced by the *kan* marker (at the end closer to a centromere) and the other by the *URA3* marker (at the end closer to a telomere).

**Fig. 5.**
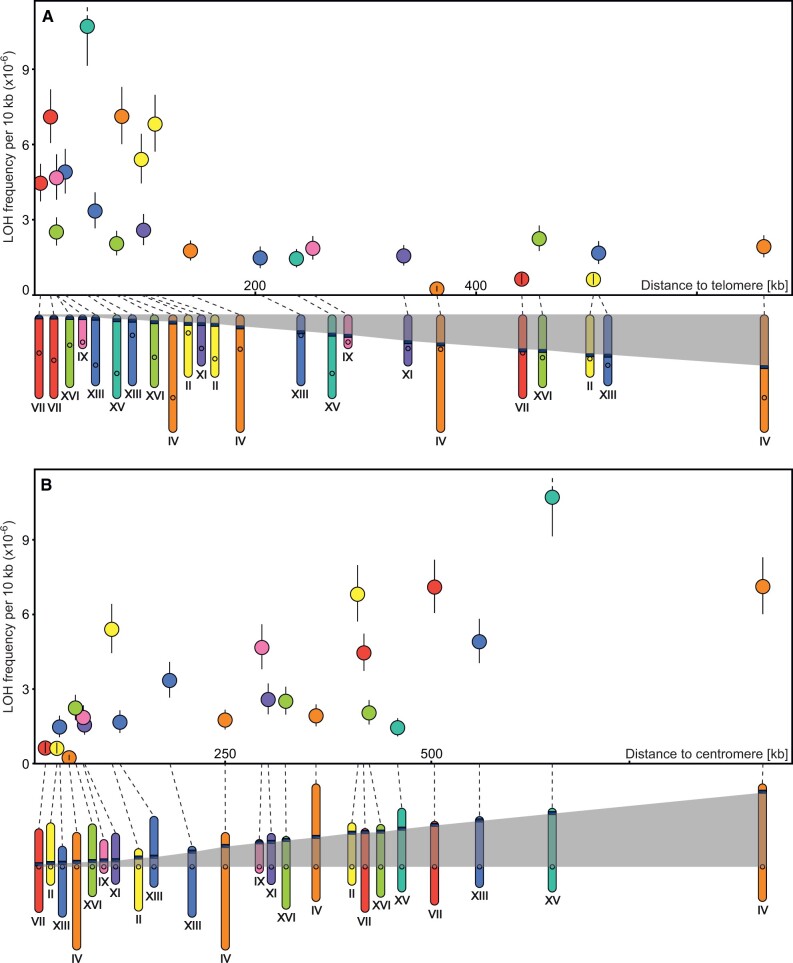
Incidence of LOH in the collection of 23 strains marked at variable positions in a subset of chromosomes. Portions of chromosomes colored in dark blue identify segments of about 40 kb bordered by *kan* (resistance to geneticin, the end toward a centromere) and *URA3* (sensitivity to 5-FOA, the end toward a telomere), circles represent centromeres. Estimates of the LOH rate were obtained after selection on medium containing both geneticin and 5-FOA. a) Segments ordered according to their proximity to a telomere (Spearman’s rank coefficient *r*_s_ = −0.714; *n = *23; *P = *0.00019). b) The same segments ordered according to their proximity to a centromere (Spearman’s rank coefficient *r*_s_ = 0.708; *n = *23; *P = *0.00023).

A final collection was composed of 23 chromosomal segments of about 40 kb that were marked on both ends as planned and located at different distances to either centromeres or telomeres. They were then assayed for LOH on the FG (5-FOA and geneticin) medium. Results are summarized in Fig. 5. The 2 graphs show the LOH rate estimates for the same set of segments but arranged in 2 different ways, according to their distance to either a telomere or a centromere. Both criteria appear to matter. Events of the within-segment recombination (or truncation) were up to several-fold more frequent in regions close to telomeres. Not necessarily very close, however, as the unstable regions spanned for tens of thousands of base pairs (Fig. 5a). Regarding centromeres, an equally strong, but opposite effect was seen. It was most visible for the LOH target sites located especially close to that end of an arm (Fig. 5b). The identity of a particular chromosome, or its arm, did not appear to be of importance.

This experiment applied a classic fluctuation test in which entire mutation-accumulating cultures were plated on a selective medium, all emerging colonies were counted, and the mutation rates were calculated with an ML method. Such a workload would be hardly possible for all the described above (and below) experiments involving the other strain collections (Fig. 1). In those experiments, only small portions of cultures were plated and the mutation rate was calculated differently. It is worth noting that both approaches yielded similar results and thus validated each other. This can be seen by comparing the *Y*-axes in Figs. 3 and 5. (Regions close to telomeres are associated with very high rates but were assayed only in this experiment and the high scores obtained for them do not appear in assays involving the collection of 32 BY/BY strains.)

### LOH is infrequent but detectable in nongrowing cells

**Fig. 6.**
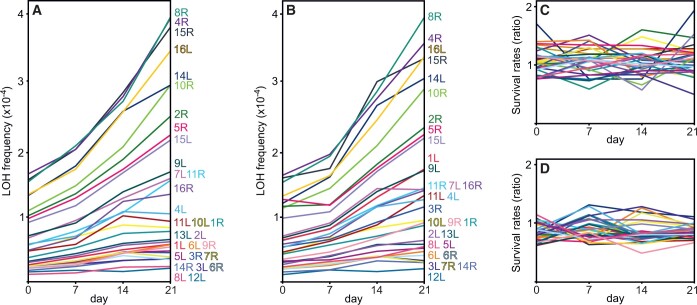
LOH in nongrowing (starving) cells. Proportion of LOH mutants among alive cells when the mutants were recovered on the F selective medium (a) or on the FG medium (b). The 2 further graphs demonstrate that the observed increase in mutant frequency was not caused by their better survival or higher plating efficiency on selective media. c) Survival rates of the LOH mutants divided by the survival rates of their progenitor strains remained constant over the course of experiment. d) Survival of the LOH mutants alone when tested on the selective FG vs nonselective medium (ratio of counts) also remained unchanged. The observed smoothness of lines shown in (a) and (b) when compared with (c) and (d) resulted from a much higher number of replicates, 30 vs 3, respectively (see *Materials and methods*).

We also asked how intense can LOH be in nongrowing cells. In this experiment, we used the original collection of 32 marked BY/BY strains. We diluted stationary phase cultures in water 1:10, let them adjust to these starvation conditions, and then sampled them 4 times. Setting the initial populations’ sizes at 1, the proportion of viable cells after 7, 14, and 21 days was 0.824, 0.499, and 0.332, respectively. Thus, the effect of starvation was considerable but not extreme, as planned. Most importantly, we found that the frequency of the LOH mutants among viable cells tended to increase over time. Figure 6, a and b demonstrates that the increase was remarkably universal and regular: (1) it was seen on both the FG and F selective media; (2) all tested strains accumulated LOH mutants; and (3) the ranking of chromosomal arms, according to their LOH frequency, remained nearly unchanged through starvation. This pattern suggests the gradual accumulation of new mutants in all strains over the entire period of starvation. One alternative (although rather unlikely) explanation could be that the LOH mutants tended to survive better than the original cells. Figure 6c shows that this was not the case. Another explanation, again highly hypothetical, would be that the selective drugs added to medium tended to uphold plating efficiency of the starving LOH mutants, and increasingly so with the passing time. Figure 6d shows that there was no such effect. (The visual perception of a lack of change in proportions over time in Fig. 6, c and d was confirmed by formal statistical analysis, Supplementary Table 7.) For all these reasons, we believe that we observed genuinely new LOH mutants emerging over the time of starvation. Whether this meant that the starving cell underwent complete LOH or rather accumulated alterations that resulted in LOH upon restarting growth remains unknown. Such a distinction is not necessary to ask about the potential impact of LOH in nongrowing cells on the genetic structure of yeast populations. To this end, we regressed the frequencies of mutants in every strain over time with a unit period of 2 h. The resulting estimate was equal to the chance that a single nongrowing cell turned into a mutant one over the time required for a cell doubling (roughly 2 h under standard conditions). We summed 30 estimates for individual between-marker stretches (the right arm of chromosome XII was excluded deliberately, the right arm of chromosome XIII was lost accidentally) and compared the result with its analog derived for the growing BY/BY. This led us to a conclusion that the nongrowing cells experienced LOH at a considerably lower rate, 26.2 times or 26.4 times per unit time, depending on whether the F or FG medium was used for screening mutants.

## Discussion

We placed selectable loci at every chromosome arm in several yeast strains and then measured the rate of phenotypically detectable LOH in several environments. This enabled us to discover several new aspects of LOH in yeast that were not detected in the older phenotype-based studies and would be difficult or sometimes impossible to find in the newer sequence-based ones. One basic question about LOH is, obviously, its frequency. Early studies used a single marker locus to address this matter. For example, it has been found that crossing-over between *CAN1* and a corresponding centromere occurs at a rate of 1.2 or 1.4 × 10^−5^ per mitotic cell division (Hartwell and Smith 1985; Klein 2001). The stretch of DNA on which these cross-overs happen lies on the short arm of chromosome V and constitutes about 1% of all nuclear DNA. Therefore, inferred genomic rates would be 1.21 × 10^−3^ and 1.41 × 10^−3^, respectively. Two other studies have focused on different single loci located on the long arm of chromosome IV. The derived estimates of the genomic rate of LOH through crossing-over have been found noticeably close to each other, 6.8 × 10^−4^ or 6.2 × 10^−4^, but about 2 times lower than those reported for *CAN1* (Andersen *et al.* 2008; Charles and Petes 2013). The *MET15* locus (on the long arm of chromosome XII) has suggested a considerably higher estimate of the genomic rate of crossing-over, 6.2 × 10^−3^ (Andersen *et al.* 2008). As those studies have not involved hybrids, their results should be compared with that of ours which relate to the homozygous BY/BY. Specifically, the value of 1.22 × 10^−3^, derived in a way described in *Results*. It is closer to the lower bound of the just mentioned estimates, which is understandable as it was based on the inner parts of chromosomes and did not account for the right arm of chromosome XII which contains the rDNA region and is known for an outstandingly high rate of homologous recombination. The observation that the rates of LOH are high on the short arm of chromosome V and low on the long arm of chromosome IV fits the reported here general pattern of negative correlation between the length of a chromosomal arm and density of mitotic recombination. More recent studies, those starting with long-term MA, have sought to estimate the genomic rate of LOH (in hybrids, by design) through analysis of DNA sequence. Most studies of this type have found the genomic rate of terminal LOH to be between 4 × 10^−4^ and 2.8 × 10^−3^ (O’Connell *et al.* 2015; Dutta *et al.* 2017; James *et al.* 2019; Pankajam *et al.* 2020; Sui *et al.* 2020). In one experiment, in which multiple hybrid strains were used to accumulate mutations, the rate of terminal LOH varied from 1.5 × 10^−3^ to 2.5 × 10^−2^. The latter estimate is higher than typically met. It may mean that the mitotic stability of the yeast genome is considerably lower in some strains (Dutta *et al.* 2021). All estimates presented in our study, 1 for homozygous and 4 for hybrid strains, overlap with those reported by most of the MA studies. A cautionary note should be added. We worked with data obtained for the inner halves of chromosomes. The figures would be somewhat higher if the more unstable outer regions were accounted for. However, it appears that any final estimate of the terminal LOH rate would be augmented by just a fraction, and not very large, of its present value.

An important advantage of the method used here was its applicability to a homozygous strain. BY/BY had the highest rate of LOH, nearly 2 times as high as that of some hybrids. This strain had also the highest excess of LOH on the long arm of chromosome XII, suggesting that strict homozygosity boosts homologous recombination in the rDNA region (Fig. 2). These observations imply that even relatively small sequence divergence may increase the proportion of unsuccessful resolutions of recombination events and thus lower the chance of postmitotic survival. Such complications are known to happen when the cell division is meiotic and sequence divergence large (Greig *et al.* 2003; Liti *et al.* 2006; Bozdag *et al.* 2021). Although we believe that the demonstrated here negative correlation between the sequence divergence and the rate of LOH can be of general applicability, we note that a seemingly opposite result was reported, i.e. the terminal LOH was more frequent in highly divergent hybrids (Dutta *et al.* 2021). It was actually true for some of the most heterozygous strains only, any overall tendency would be hard to discern. Still, claims about generality of either of the opposite patterns need to be made carefully.

We found that the average rate of LOH was considerably higher for shorter chromosomal arms. In an independent experiment, we demonstrated that the rate of LOH could be high also on long chromosomal arms but in regions close to their telomeres. The segments of an enhanced LOH appeared to be fairly large. They possibly extended over distances no smaller than halves of the short chromosomal arms and this could be why the average rate of LOH was higher for them. If so, it would mean that the proximity of a telomere was more consequential than the proximity of a centromere for the final LOH rate on short arms. It is not obvious what particular mechanism(s) was responsible for that. The simplest explanation would be that the density of initial damages to DNA is highly uneven. It cannot be excluded that they were more frequent close to telomeres even though the regions studied by us were usually separated from the intensely processed stretches of repeated fragments by at least several coding sequences. On the other hand, sequences adjacent to a centromere might be more protected after adopting more compact forms of chromatin. Alternatively, it is possible that the incidence of DNA damage was rather homogeneous but the efficiency or mode of DNA repair varied systematically. For example, damages in terminal regions might have been more often repaired with a nonsister template which is a prerequisite for a recombination event to be detectable. Even a relatively small bias would be consequential because typically sister chromatids provide repair templates at least 10 times more frequently than nonsister ones (Jinks-Robertson and Petes 2021). Repairs through break-induced recombination are believed to be considerably less frequent than those leading to cross-overs or gene conversions (Pham *et al.* 2021) but they appear to be easier to complete when the end of a chromosome is near and this could also contribute to the observed pattern. Finally, it is possible that repairs were relatively often not finished and a progeny cell received a truncated chromosome. Such a cell would be less harmed by a negative effect of haploinsufficiency if the lacking segment was short. That is, truncation of a shorter arm would not be punished severely by natural selection, providing a chance for an eventual repair before one of the next divisions. All these scenarios are admittedly only hypothetical. However, considering them all may help to interpret existing data and advise on future research.

We also tested for possible associations between the rate of LOH and the distribution of repetitive genetic elements. In choosing these elements, we followed previous studies reporting that such associations have been at least sometimes seen (Rattray and Symington 1993; Aguilera *et al.* 2000; Gelfand *et al.* 2007; Mancera *et al.* 2008; Chan and Kolodner 2011; Symington *et al.* 2014). In those studies, however, the elements promoting destabilization were typically close to the loci serving to detect it. In our study, the potentially destabilizing elements were placed on a long tract of DNA, between a centromere and the center of a chromosome arm, and assumed to contribute their effects additively. Therefore, it is worth noting that some of the searched associations were detectable still, demonstrating that the impact of repetitive elements on LOH could be felt on the scale of whole chromosome arms. The positive correlation with the density of meiotic hotspots is an ambiguous result. On the one hand, they might contribute by accidental initiation of recombination in mitosis. On the other, they have to be more densely distributed on shorter arms to ensure initiation of chiasmatic connections there, and thus the uncovered by us relation could be spurious (Murakami *et al.* 2020).

Another possible LOH factor is transcription intensity (Aguilera 2002). In our study, there were several-fold differences between chromosomal arms and environments in the amount of RNA transcribed over a unit length of DNA. If this activity affected the rate of LOH as effectively as the presence of repetitive elements, a statistically significant correlation would likely emerge. One explanation of a nonsignificant result could be that the interfering effect of transcription is restricted mostly to the time of DNA synthesis. It has been reported that the RNA-transcribing complex can interact with the 1 replicating DNA and that such encounters elevate the rate of LOH (Liu *et al.* 2021). In our experiment, mRNA transcripts were collected from cells undergoing all phases of the cell cycle and those specific for the S phase might have been overcrowded by other ones. Our findings may be interpreted as supporting the view that the separation of strands and then adoption of one of them as a template for transcription are in themselves neutral for LOH as long as they proceed undisturbed by, for example, replication of DNA.

Both point mutations and chromosomal mutations are typically seen as resulting from inaccurate replication and division of genetic material. This could suggest that both phenomena are confined to the process of cell proliferation. In the case of mutation, it has been shown that accumulation of mutations over many generations, known as a molecular clock, cannot be explained only by the number of cell divisions but also the average cell lifespan (Gao *et al.* 2016, 2019). There are also experimental studies, including those using the budding yeast, reporting that cells undergoing quiescence/starvation accumulate mutations at enhanced rates and with possibly altered spectra (Greene and Jinks-Robertson 1999; Heidenreich and Wintersberger 2001; Halas *et al.* 2009; Skoneczna *et al.* 2015).

Regarding chromosomal mutations, it has been reported that the rate of different forms of aneuploidization can increase by orders of magnitude in starving yeast cells (Coyle and Kroll 2008; Kroll *et al.* 2013). We do not see it in this experiment where both recombination and loss of chromosomes could happen. In our system, LOH does happen in nongrowing yeast cells, although at a much lower rate than in growing ones, when measured per time unit. It appears that starvation does not abolish mechanisms defending the stability of the genome. In yeast, absence or severe limitation of nutrients leads to specific cellular reactions which are similar to those stimulated by different environmental stresses and include condensation of chromatin (Klosinska *et al.* 2011). Thus, the starving yeast cell does protect its integrity and this may be the reason why the observed rate of LOH was generally low. It was so low that any study based on DNA analysis would require sequencing thousands of cells to detect a single LOH which makes such an approach unworkable. Our results can be used to interpret data on LOH in other yeast strains that may undergo very different periods of growth and starvation. One cautionary note is that this particular strain (BY), especially under our protocol, was virtually guaranteed not to enter sporulation after glucose was depleted. Wild strains do it much more readily. Furthermore, an influx of resources into a cell that initiated meiosis may result in a return-to-growth development that skirts meiosis but results in unusually frequent recombination (Laureau *et al.* 2016). Thus, although starvation itself does not promote LOH, frequent onsets of starvation, sporulation, and growth reassumption may be responsible for it.

In general, our study provided amounts of data large enough to demonstrate the existence of genome-wide patterns in LOH: its rate increases when the number of SNPs between homologous chromosomes decreases and is higher on shorter chromosomes, or more generally, in regions relatively close to telomeres. It helped to reveal which repetitive elements can alter the pattern of LOH not only locally but also at a scale of whole chromosomal arms. It also demonstrated that the unfavorable conditions of starvation tend to substantially decrease and not increase the overall rate of LOH. Thus, methods relying on phenotypic detection of LOH remain useful and should be used along those implementing intense sequencing of DNA.

## Data availability

Strains are available upon request. The data underlying this article are shown in the article and in its online supplementary material except for the mRNA expression data which are available at the GEO (Gene Expression Omnibus) under the number GSE192479.

Supplemental material is available at *GENETICS* online.

## Supplementary Material

iyac032_Supplemental_Table_1

iyac032_Supplemental_Table_2

iyac032_Supplemental_Table_3

iyac032_Supplemental_Table_4

iyac032_Supplemental_Table_5

iyac032_Supplemental_Table_6

iyac032_Supplemental_Table_7
